# Supplementary material for: Novel brain biomarkers of obesity in young adult women based on statistical measurements of white matter tracts
Source: PLoS One. 2025 Apr 10;20(4):e0319936. doi: 10.1371/journal.pone.0319936 (PMC11984704; doi:10.1371/journal.pone.0319936)
Supplement: S1 Fig — Boxplots for the Dnorm (blue) and Dover (red) sets, with the white matter tract indicated on the horizontal axis and the value of the measured statistic on the vertical axis. Only the tracts and measurements that had statistically significant differences (pw < 0.05) after applying the nonparametric Wilcoxon rank sum test, comparing the subjects of the Dnorm and Dover sets, are presented. These tracts and measurements are the same ones that obtained significant Spearman correlations (pc < 0.05, FDR-corrected) with the BMI values of all the study subjects of the Dall set, which can be observed in Fig 2. (PDF) [file pone.0319936.s001.pdf]

S1 Fig. Boxplots of the tract measurements studied. Boxplots for the D_{norm} (blue) and D_{over} (red) sets, with the white matter tract indicated on the horizontal axis and the value of the measured statistic on the vertical axis. Only the tracts and measurements that had statistically significant differences ($p_w < 0.05$) after applying the nonparametric Wilcoxon rank sum test, comparing the subjects of the D_{norm} and D_{over} sets, are presented. These tracts and measurements are the same ones that obtained significant Spearman correlations ($p_c < 0.05$, FDR-corrected) with the BMI values of all the study subjects of the D_{all} set, which can be observed in Fig. 2.

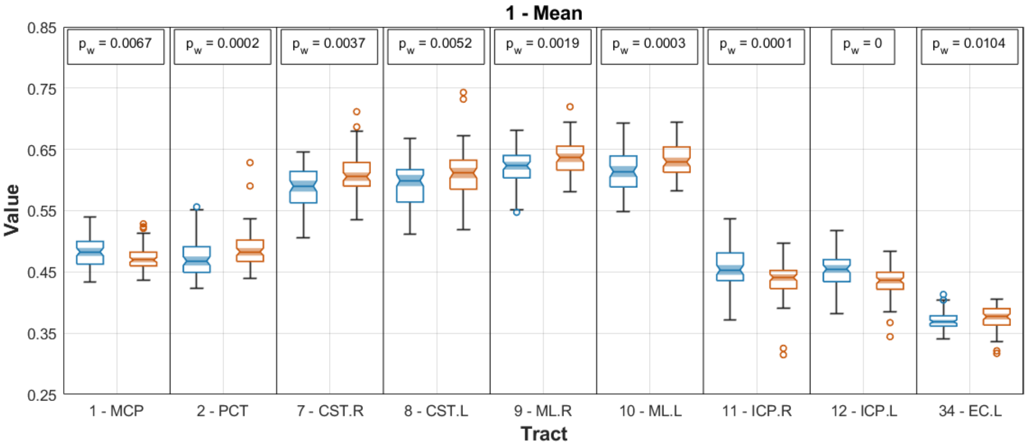

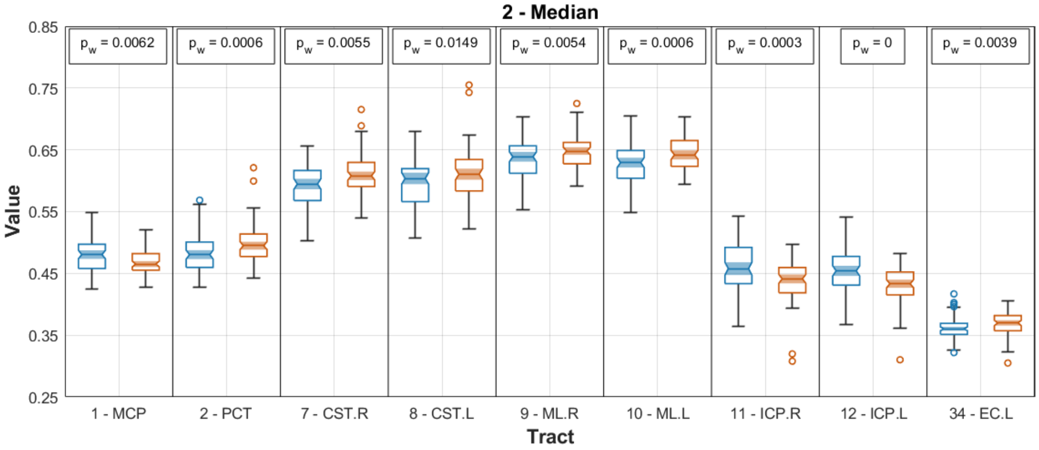

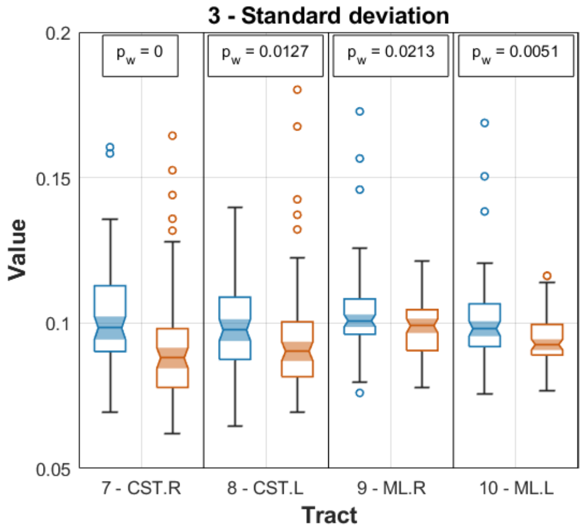
3 - Standard deviation
Value
Tract
pw = 0
pw = 0.0127
pw = 0.0213
pw = 0.0051
7 - CST.R
8 - CST.L
9 - ML.R
10 - ML.L

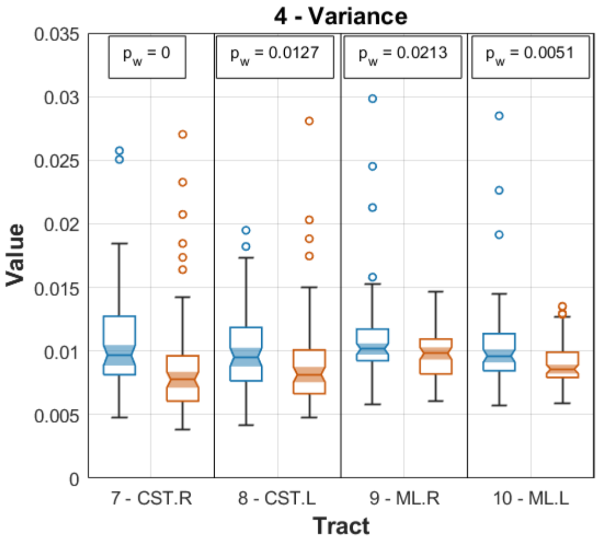
4 - Variance
Value
Tract
pw = 0
pw = 0.0127
pw = 0.0213
pw = 0.0051
7 - CST.R
8 - CST.L
9 - ML.R
10 - ML.L

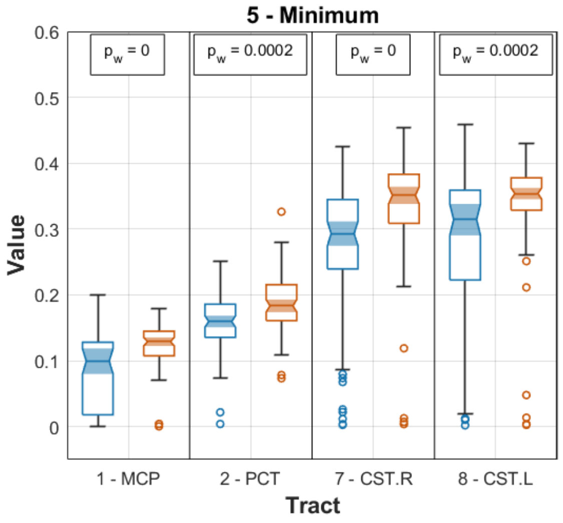
5 - Minimum
Value
Tract
pw = 0
pw = 0.0002
pw = 0
pw = 0.0002
1 - MCP
2 - PCT
7 - CST.R
8 - CST.L

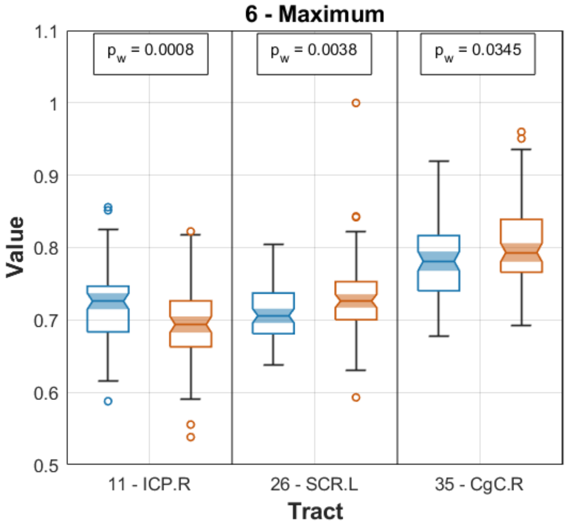
6 - Maximum
Value
Tract
pw = 0.0008
pw = 0.0038
pw = 0.0345
11 - ICP.R
26 - SCR.L
35 - CgC.R

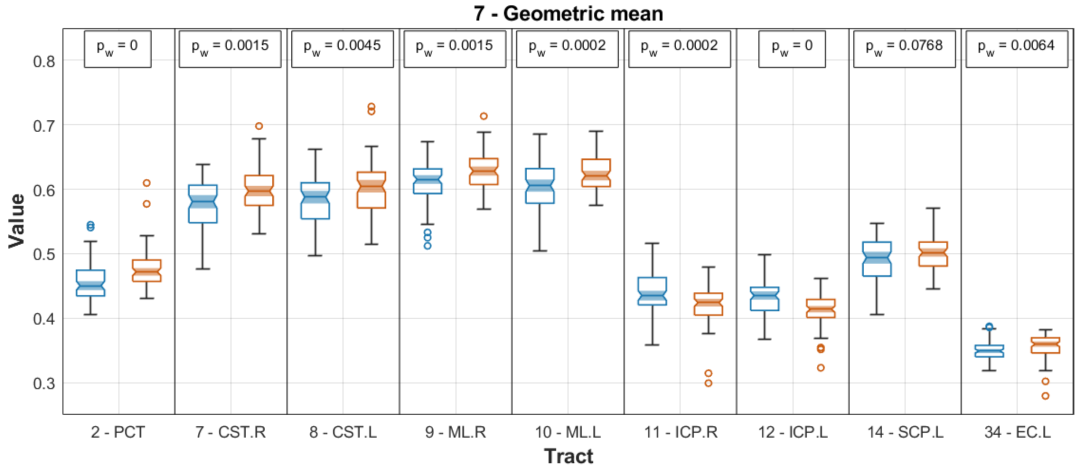
7 - Geometric mean
p_w = 0
p_w = 0.0015
p_w = 0.0045
p_w = 0.0015
p_w = 0.0002
p_w = 0.0002
p_w = 0
p_w = 0.0768
p_w = 0.0064
Value
0.8
0.7
0.6
0.5
0.4
0.3
2 - PCT
7 - CST.R
8 - CST.L
9 - ML.R
10 - ML.L
11 - ICP.R
12 - ICP.L
14 - SCP.L
34 - EC.L
Tract

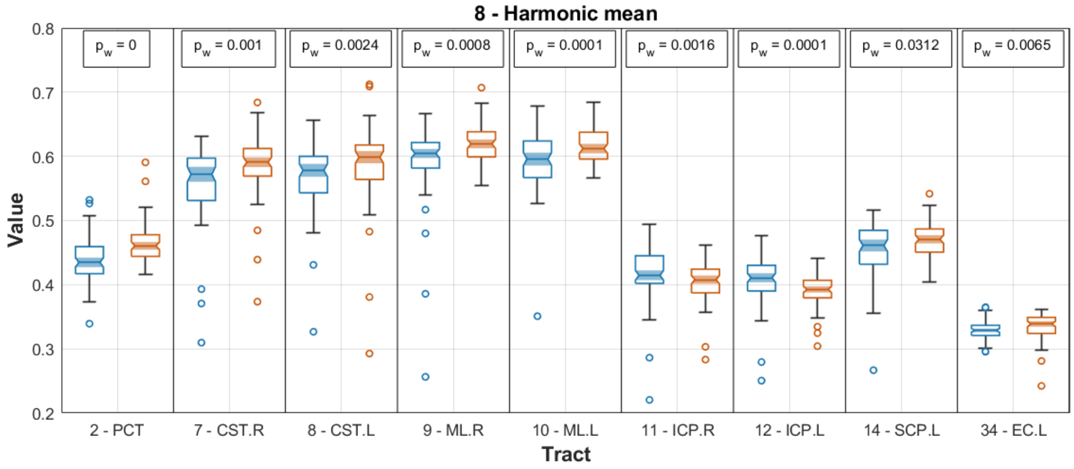
8 - Harmonic mean
p_w = 0
p_w = 0.001
p_w = 0.0024
p_w = 0.0008
p_w = 0.0001
p_w = 0.0016
p_w = 0.0001
p_w = 0.0312
p_w = 0.0065
Value
0.8
0.7
0.6
0.5
0.4
0.3
0.2
2 - PCT
7 - CST.R
8 - CST.L
9 - ML.R
10 - ML.L
11 - ICP.R
12 - ICP.L
14 - SCP.L
34 - EC.L
Tract

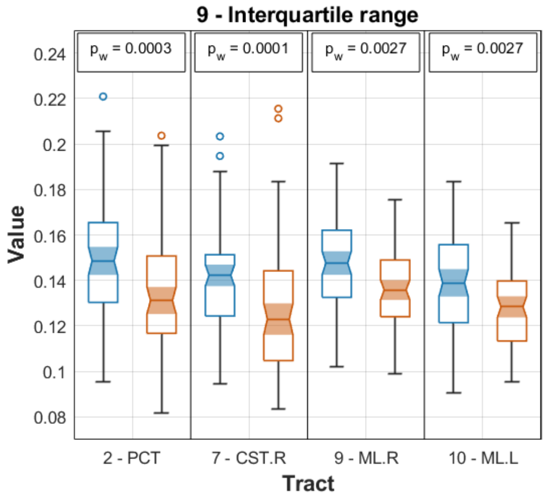
9 - Interquartile range
p w = 0.0003
p w = 0.0001
p w = 0.0027
p w = 0.0027
0.24
0.22
0.2
0.18
0.16
0.14
0.12
0.1
0.08
Value
2 - PCT
7 - CST.R
9 - ML.R
10 - ML.L
Tract

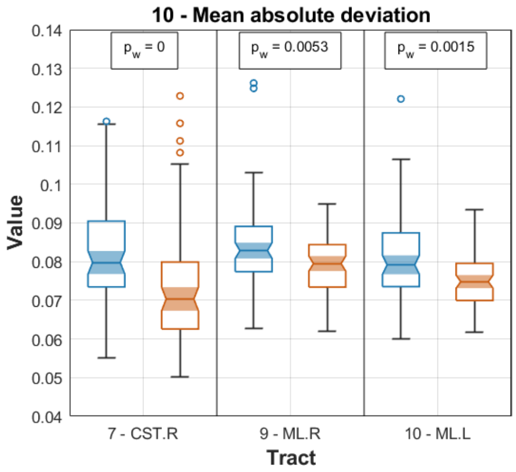
10 - Mean absolute deviation
p w = 0
p w = 0.0053
p w = 0.0015
0.14
0.13
0.12
0.11
0.1
0.09
0.08
0.07
0.06
0.05
0.04
Value
7 - CST.R
9 - ML.R
10 - ML.L
Tract

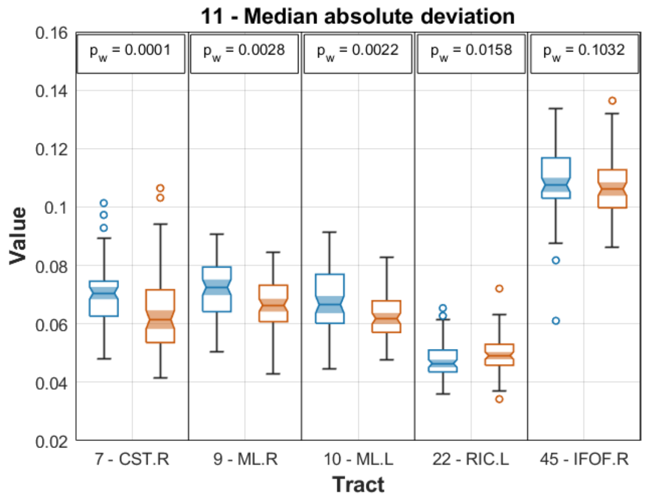

11 - Median absolute deviation
Value
Tract
0.16
0.14
0.12
0.1
0.08
0.06
0.04
0.02
p_w = 0.0001
p_w = 0.0028
p_w = 0.0022
p_w = 0.0158
p_w = 0.1032
7 - CST.R
9 - ML.R
10 - ML.L
22 - RIC.L
45 - IFOF.R

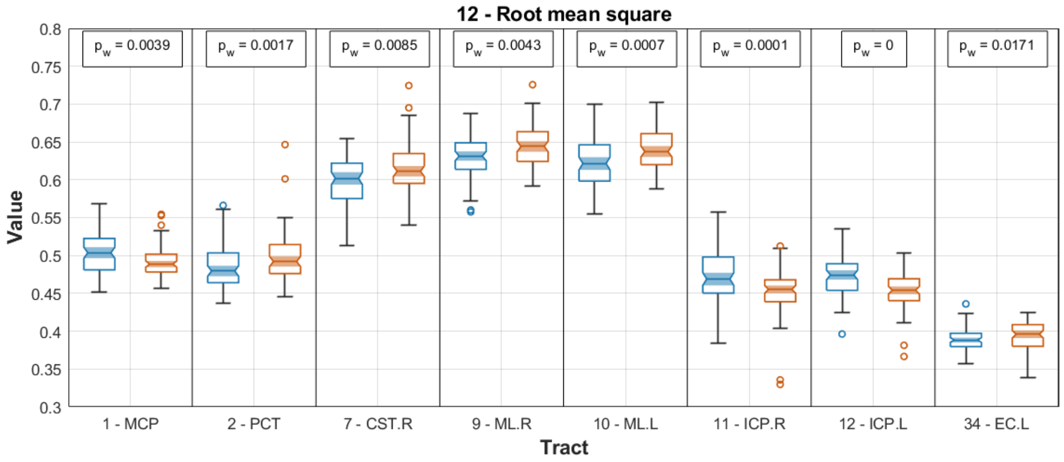

12 - Root mean square
Value
Tract
0.8
0.75
0.7
0.65
0.6
0.55
0.5
0.45
0.4
0.35
0.3
p_w = 0.0039
p_w = 0.0017
p_w = 0.0085
p_w = 0.0043
p_w = 0.0007
p_w = 0.0001
p_w = 0
p_w = 0.0171
1 - MCP
2 - PCT
7 - CST.R
9 - ML.R
10 - ML.L
11 - ICP.R
12 - ICP.L
34 - EC.L
