# Supplementary material for: Novel brain biomarkers of obesity in young adult women based on statistical measurements of white matter tracts
Source: PLoS One. 2025 Apr 10;20(4):e0319936. doi: 10.1371/journal.pone.0319936 (PMC11984704; doi:10.1371/journal.pone.0319936)
Supplement: S2 Fig — For the training, validation and testing subsets, 80, 40 and 40 subjects respectively were randomly selected. An approximate 2:1 ratio was sought between subjects with the same BMI value in the training subset and the validation or testing subsets. (PDF) [file pone.0319936.s002.pdf]

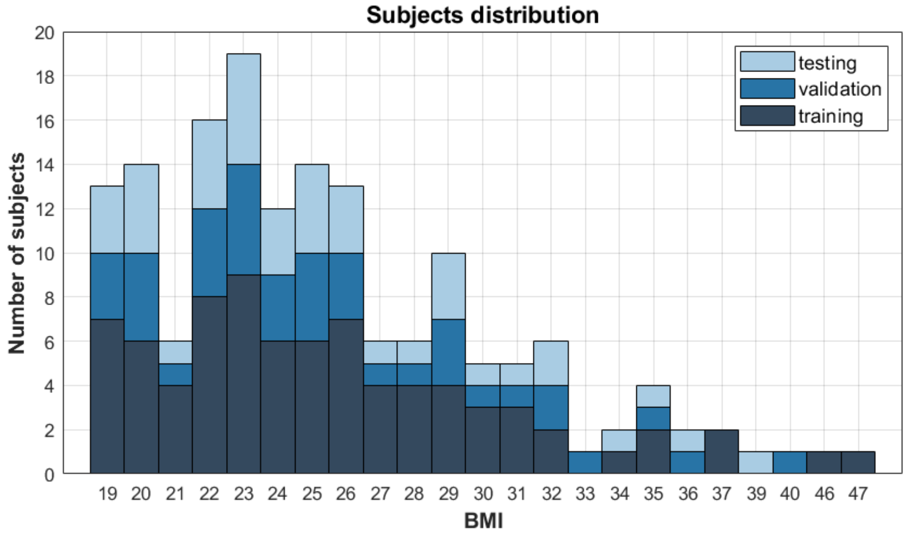

S2 Fig. Distribution of subjects by subset and BMI. For the training, validation and testing subsets, 80, 40 and 40 subjects respectively were randomly selected. An approximate 2:1 ratio was sought between subjects with the same BMI value in the training subset and the validation or testing subsets.
