# Supplementary material for: Novel brain biomarkers of obesity in young adult women based on statistical measurements of white matter tracts
Source: PLoS One. 2025 Apr 10;20(4):e0319936. doi: 10.1371/journal.pone.0319936 (PMC11984704; doi:10.1371/journal.pone.0319936)
Supplement: S1 Table — Participant IDs provided in the ID1000 database. (PDF) [file pone.0319936.s003.pdf]

sub-0001	sub-0089	sub-0170	sub-0308	sub-0422	sub-0489	sub-0636	sub-0712
sub-0002	sub-0091	sub-0192	sub-0309	sub-0425	sub-0515	sub-0640	sub-0727
sub-0006	sub-0094	sub-0204	sub-0354	sub-0429	sub-0536	sub-0644	sub-0804
sub-0037	sub-0095	sub-0216	sub-0360	sub-0431	sub-0539	sub-0664	sub-0811
sub-0047	sub-0104	sub-0226	sub-0363	sub-0443	sub-0547	sub-0671	sub-0833
sub-0072	sub-0118	sub-0254	sub-0368	sub-0446	sub-0566	sub-0687	sub-0834
sub-0079	sub-0127	sub-0264	sub-0374	sub-0448	sub-0578	sub-0691	sub-0849
sub-0084	sub-0141	sub-0279	sub-0384	sub-0458	sub-0579	sub-0700	sub-0875
sub-0086	sub-0145	sub-0286	sub-0385	sub-0467	sub-0617	sub-0709	sub-0882
sub-0088	sub-0169	sub-0291	sub-0412	sub-0477	sub-0628	sub-0711	sub-0917

S1 Table. Subjects with normal weight studied in this work. Participant IDs provided in the ID1000 database.
