# Supplementary material for: Novel brain biomarkers of obesity in young adult women based on statistical measurements of white matter tracts
Source: PLoS One. 2025 Apr 10;20(4):e0319936. doi: 10.1371/journal.pone.0319936 (PMC11984704; doi:10.1371/journal.pone.0319936)
Supplement: S2 Table — Participant IDs provided in the ID1000 database. (PDF) [file pone.0319936.s004.pdf]

sub-0008	sub-0133	sub-0221	sub-0298	sub-0451	sub-0572	sub-0764	sub-0850
sub-0020	sub-0138	sub-0236	sub-0301	sub-0483	sub-0582	sub-0774	sub-0857
sub-0026	sub-0143	sub-0243	sub-0302	sub-0487	sub-0591	sub-0775	sub-0859
sub-0040	sub-0154	sub-0259	sub-0312	sub-0494	sub-0643	sub-0794	sub-0862
sub-0064	sub-0155	sub-0260	sub-0342	sub-0495	sub-0646	sub-0809	sub-0866
sub-0099	sub-0162	sub-0278	sub-0357	sub-0499	sub-0649	sub-0818	sub-0870
sub-0121	sub-0165	sub-0283	sub-0381	sub-0508	sub-0666	sub-0826	sub-0886
sub-0123	sub-0177	sub-0285	sub-0404	sub-0525	sub-0688	sub-0827	sub-0893
sub-0130	sub-0187	sub-0292	sub-0405	sub-0535	sub-0726	sub-0845	sub-0902
sub-0131	sub-0190	sub-0297	sub-0437	sub-0563	sub-0753	sub-0847	sub-0927

S2 Table. Subjects with overweight/obesity studied in this work. Participant IDs provided in the ID1000 database.
