# Supplementary material for: Novel brain biomarkers of obesity in young adult women based on statistical measurements of white matter tracts
Source: PLoS One. 2025 Apr 10;20(4):e0319936. doi: 10.1371/journal.pone.0319936 (PMC11984704; doi:10.1371/journal.pone.0319936)
Supplement: S3 Table — White matter parcellation. (PDF) [file pone.0319936.s005.pdf]

Index	Tract	Abbreviation	Category
1	Middle cerebellar peduncle	MCP	Brainstem
2	Pontine crossing tract	PCT	Brainstem
3	Genu of corpus callosum	gCC	Commissural
4	Body of corpus callosum	bCC	Commissural
5	Splenium of corpus callosum	sCC	Commissural
6	Fornix (column and body of fornix)	FX	Association
7	Corticospinal tract R	CST.R	Brainstem
8	Corticospinal tract L	CST.L	Brainstem
9	Medial lemniscus R	ML.R	Brainstem
10	Medial lemniscus L	ML.L	Brainstem
11	Inferior cerebellar peduncle R	ICP.R	Brainstem
12	Inferior cerebellar peduncle L	ICP.L	Brainstem
13	Superior cerebellar peduncle R	SCP.R	Brainstem
14	Superior cerebellar peduncle L	SCP.L	Brainstem
15	Cerebral peduncle R	CP.R	Projection
16	Cerebral peduncle L	CP.L	Projection
17	Anterior limb of internal capsule R	ALIC.R	Projection
18	Anterior limb of internal capsule L	ALIC.L	Projection
19	Posterior limb of internal capsule R	PLIC.R	Projection
20	Posterior limb of internal capsule L	PLIC.L	Projection
21	Retrolenticular part of internal capsule R	RIC.R	Projection
22	Retrolenticular part of internal capsule L	RIC.L	Projection
23	Anterior corona radiata R	ACR.R	Projection
24	Anterior corona radiata L	ACR.L	Projection
25	Superior corona radiata R	SCR.R	Projection
26	Superior corona radiata L	SCR.L	Projection
27	Posterior corona radiata R	PCR.R	Projection
28	Posterior corona radiata L	PCR.L	Projection
29	Posterior thalamic radiation R	PTR.R	Projection
30	Posterior thalamic radiation L	PTR.L	Projection
31	Sagittal stratum R	SS.R	Association
32	Sagittal stratum L	SS.L	Association
33	External capsule R	EC.R	Association
34	External capsule L	EC.L	Association
35	Cingulum (cingulate gyrus) R	CgC.R	Association
36	Cingulum (cingulate gyrus) L	CgC.L	Association
37	Cingulum (hippocampus) R	CgH.R	Association
38	Cingulum (hippocampus) L	CgH.L	Association
39	Fornix (cres) / Stria terminalis R	FX/ST.R	Association
40	Fornix (cres) / Stria terminalis L	FX/ST.L	Association
41	Superior longitudinal fasciculus R	SLF.R	Association
42	Superior longitudinal fasciculus L	SLF.L	Association
43	Superior fronto-occipital fasciculus R	SFOF.R	Association
44	Superior fronto-occipital fasciculus L	SFOF.L	Association
45	Inferior fronto-occipital fasciculus R	IFOF.R	Association
46	Inferior fronto-occipital fasciculus L	IFOF.L	Association
47	Uncinate fasciculus R	UF.R	Association
48	Uncinate fasciculus L	UF.L	Association
49	Tapetum R	TAP.R	Commissural
50	Tapetum L	TAP.L	Commissural

S3 Table. ICBM-DTI-81 white-matter labels atlas. White matter parcellation.
