# Supplementary material for: Novel brain biomarkers of obesity in young adult women based on statistical measurements of white matter tracts
Source: PLoS One. 2025 Apr 10;20(4):e0319936. doi: 10.1371/journal.pone.0319936 (PMC11984704; doi:10.1371/journal.pone.0319936)
Supplement: S4 Table — For each tract, 12 descriptive statistics were calculated from the voxel values considering the FA images. (PDF) [file pone.0319936.s006.pdf]

Measurement	Equation	Measurement	Equation
M_1: Mean	$\mu(A) = \frac{1}{N}\sum_{i=1}^{N} A_i$	M_7: Geometric mean	$GM(A) = \left[\prod_{i=1}^{N} A_i\right]^{\frac{1}{N}}$
M_2: Median	$\tilde{\mu}(A)$	M_8: Harmonic mean	$HM(A) = N \Big/ \sum_{i=1}^{N} \frac{1}{A_i}$
M_3: Standard desviation	$\mathrm{SD}(A) = \sqrt{\frac{1}{N-1}\sum_{i=1}^{N} \lvert A_i - \mu(A)\rvert^2}$	M_9: Interquartile range	$IQ(A) = Third\ quartile(A) - first\ quartile(A)$
M_4: Variance	$\mathrm{VAR}(A) = \frac{1}{N-1}\sum_{i=1}^{N} \lvert A_i - \mu(A)\rvert^2$	M_{10}: Mean absolute deviation	$MAD(A) = \mu(\lvert A_i - \mu(A)\rvert)$
M_5: Minimum	$min(A)$	M_{11}: Median absolute deviation	$MDAD(A) = \tilde{\mu}(\lvert A_i - \tilde{\mu}(A)\rvert)$
M_6: Maximum	$max(A)$	M_{12}: Root mean square value	$RMS(A) = \sqrt{\frac{1}{N}\sum_{i=1}^{N} \lvert A_i\rvert^2}$

S4 Table. Tract measurements. For each tract, 12 descriptive statistics were calculated from the voxel values considering the FA images.
