# Supplementary material for: Novel brain biomarkers of obesity in young adult women based on statistical measurements of white matter tracts
Source: PLoS One. 2025 Apr 10;20(4):e0319936. doi: 10.1371/journal.pone.0319936 (PMC11984704; doi:10.1371/journal.pone.0319936)
Supplement: S5 Table — Details on published papers that have reported correlations between WM integrity and obesity. (PDF) [file pone.0319936.s007.pdf]

| Study | Subjects (Female) | Age (years) | BMI (kg/m²) | DTI analysis |
|---|---|---|---|---|
| Best et al. 2020 [42] | 1065 (575) | 28.75 ± 3.67 | 26.40 ± 5.11 | TBSS |
| Birdsill et al. 2017 [11] | 168 (96) | 49.5 ± 6.4 | 29.8 ± 6.7 | TBSS |
| Dietze et al. 2023 [7] | 5,237 | 8 to 92 | 27.29 ± 2.91 | VBA/TBSS |
| Figley et al. 2016 [43] | 32 (16) | 18 to 57 | 18 to 37 | VBA |
| Karlsson et al. 2013 [44] | 22 (15) (N)<br>23 (18) (OV/OB) | 46.4±9.5 (N)<br>47.3±8.9 (OV/OB) | 24.0±2.3 (N) 43.2±3.7 (OV/OB) | VBA |
| Kullmann et al. 2016 [45] | 48 (23) | 21 to 36 | 19.5 to 39.3 | VBA |
| Lou et al. 2014 [46] | 22 (27) | 29.04±7.32 (N),<br>31.72±8.04 (OV/OB) | 21.54±2.06 (N) 31.44±3.34 (OV/OB) | TBSS |
| Papageorgiou et al. 2016 [47] | 120 (76) (N)<br>148 (77) (OV/OB) | 39.8 ± 15.8 (N)<br>51.5 ±14.8/<br>52.0 ± 15.7 (OV/OB) | Not reported | TBSS |
| Patel et al. 2022 [48] | 281 (128) | 60.9 ± 9.6 | 26.5 ± 4.5 | TBSS |
| Rahmani et al. 2022 [12] | 231 (124) | 69.2±8.3 | 27.3±4.7 (male) 28.2±6.6 (female) | CT |
| Repple et al. 2018 [4] | 369 (186) | 39.4 (11.2) | 24.7 (4.1) | TBSS |
| Shott et al. 2015 [49] | 24 (24) (N)<br>18 (18) (OV/OB) | 27.4±6.3 (N), 28.7±8.3 (OV/OB) | 21.64 ± 1.26 (N) 34.78 ± 4.44 (OV/OB) | VBA |
| Verstynen et al. 2012 [10] | 155 (77) | 40.7 ± 6.2 | 27.15 ± 4.82 | VBA |
| Zhang et al. 2018 [50] | 636 (322) | 55.43 ± 16.05 | 25.82 ± 3.69 | TBSS |

**S5 Table. State of the art of WM integrity vs. Obesity.** Details on published papers that have reported correlations between WM integrity and obesity.
