# Supplementary material for: Novel brain biomarkers of obesity in young adult women based on statistical measurements of white matter tracts
Source: PLoS One. 2025 Apr 10;20(4):e0319936. doi: 10.1371/journal.pone.0319936 (PMC11984704; doi:10.1371/journal.pone.0319936)
Supplement: S6 Table — The tracts that presented significant correlations (pc < 0.05, FDR-corrected) in this work, between WM integrity and BMI, are listed. The statistics mean (arithmetic), median, geometric mean and/or harmonic mean calculated on the tracts in the FA images were considered. Correlations are presented as a color scheme, positive values (blue cells) or negative values (pink cells) were used to this end. Works that reported findings in the listed tracts are also highlighted. Abbreviations used: N: normal weight; OV/OB: overweight/obese; BMI: body mass index; AFR: abdominal fat ratio; WC: waist circumference. (PDF) [file pone.0319936.s008.pdf]

WM Tract	Category	Function/connection	Statistics	Measurement	FA Correlation / Contrast (This work)	Other work	Measurement	FA Correlation or Contrast
1 - Middle cerebellar peduncle	Brainstem	Initiation, planning, and timing of volitional motor activity; posture, balance, and coordination [51].	Mean, median	BMI	Negative / N > OV/OB	Dietze et al.	BMI	Negative
						Kullmann et al.	BMI	Negative
						Verstynen et al.	BMI	Positive
						Lv et al.	Cumulative BMI	Positive
2 - Pontine crossing tract	Brainstem	Part of middle cerebellar peduncle [51].	Mean, median, geometric, armonic	BMI	Positive / N < OV/OB	Best et al.	BMI	Negative
						Verstynen et al.	BMI	Negative
						Lv et al.	Cumulative BMI	Positive
7 - Corticospinal tract R	Brainstem	Principal motor pathway for voluntary movements [27].	Mean, median, geometric, armonic	BMI	Positive / N < OV/OB	Rahmani et al.	BMI	Negative
						Lou et al.	BMI / WC	Negative
						Karlsson et al.	-	N > OV/OB
						Dietze et al.	BMI	Negative
						Verstynen et al.	BMI	Negative
						Papageorgiou et al.	-	N > OV/OB
						Lv et al.	Cumulative BMI	Positive
8 - Corticospinal tract L	Brainstem	Principal motor pathway for voluntary movements [27].	Mean, median, geometric, armonic	BMI	Positive / N < OV/OB	Rahmani et al.	BMI	Negative (male)
						Papageorgiou et al.	-	N > OV/OB
						Lou et al.	BMI / WC	Negative
						Patel et al.	AFR	Negative
						Best et al.	BMI	Negative
						Karlsson et al.	-	N > OV/OB
						Dietze et al.	BMI	Negative
						Verstynen et al.	BMI	Negative
						Rahmani et al.	BMI	Positive (female)
						Lv et al.	Cumulative BMI	Positive
9 - Medial lemniscus R	Brainstem	Convey sensations of touch, vibration, proprioception, and 2-point discrimination [51].	Mean, median, geometric, armonic	BMI	Positive / N < OV/OB	Verstynen et al.	BMI	Negative
10 - Medial lemniscus L	Brainstem	Convey sensations of touch, vibration, proprioception, and 2-point discrimination [51].	Mean, median, geometric, armonic	BMI	Positive / N < OV/OB	Verstynen et al.	BMI	Negative
11 - Inferior cerebellar peduncle R	Brainstem	Motor control such as coordination of movement control of balance, posture, and gait [52].	Mean, median, geometric, armonic	BMI	Negative / N > OV/OB	Verstynen et al.	BMI	Negative
12 - Inferior cerebellar peduncle L	Brainstem	Motor control such as coordination of movement control of balance, posture, and gait [52].	Mean, median, geometric, armonic	BMI	Negative / N > OV/OB	Verstynen et al.	BMI	Negative
14 - Superior cerebellar peduncle L	Brainstem	Motor coordination and balance network [51].	geometric, armonic	BMI	Positive / N < OV/OB	Verstynen et al.	BMI	Negative
						Zhang et al.	BMI	Negative
34 - External capsule L	Association	Carries fibers directly to the striatum from the prefrontal cortex [53].	Mean, median, geometric, armonic	BMI	Positive / N < OV/OB	Patel et al.	AFR	Negative
						Zhang et al.	BMI	Negative
						Shott et al.	-	N > OV/OB
						Repple et al.	BMI / WC	Negative
						Birdsill et al.	WC	Positive

S6 Table. Comparison of findings of this work vs. state of the art. The tracts that presented significant correlations ($p_c < 0.05$, FDR-corrected) in this work, between WM integrity and BMI, are listed. The statistics mean (arithmetic), median, geometric mean and/or harmonic mean calculated on the tracts in the FA images were considered. Correlations are presented as a color scheme, positive values (blue cells) or negative values (pink cells) were used to this end. Works that reported findings in the listed tracts are also highlighted. Abbreviations used: N: normal weight; OV/OB: overweight/obese; BMI: body mass index; AFR: abdominal fat ratio; WC: waist circumference.
