# Supplementary material for: Novel brain biomarkers of obesity in young adult women based on statistical measurements of white matter tracts
Source: PLoS One. 2025 Apr 10;20(4):e0319936. doi: 10.1371/journal.pone.0319936 (PMC11984704; doi:10.1371/journal.pone.0319936)
Supplement: S7 Table — Data is presented in the same manner as S4 Table. Abbreviations used: N: normal weight; OV/OB: overweight/obese; BMI: body mass index; AFR: abdominal fat ratio; WC: waist circunference; BFP: body fat percent. (PDF) [file pone.0319936.s009.pdf]

WM Tract	Category	Function/connection	Statistics	Measurement	FA Correlation / Contrast (This work)	Other work	Measurement	FA Correlation or Contrast
22 Retrolenticular part of internal capsule L	Projection	Contains fibers of the optic radiations [54].	Median absolute deviation	BMI	Positive / N < OV/OB	Birdsill et al.	WC	Positive
26 Superior corona radiata L	Projection	Projects from the thalamus to the sensory cortice [55].	Maximum	BMI	Positive / N < OV/OB	Figley et al.	BFP	Negative
						Patel et al.	AFR	Negative
						Shott et al.	-	N > OV/OB
						Verstynen et al.	BMI	Negative
						Birdsill et al.	WC	Positive
35 Cingulum (cingulate gyrus) R	Association	Affect, visceromotor control; response selection in skeletomotor control; visuospatial processing and memory access [51].	Maximum	BMI	Positive / N < OV/OB	Papageorgiou et al.	-	N > OV/OB
						Verstynen et al.	BMI	Negative
						Birdsill et al.	WC	Positive
45 Inferior fronto-occipital fasciculus R	Association	Integration of auditory and visual association cortices with prefrontal cortex [51].	Median absolute deviation	BMI	Negative / N > OV/OB	Rahmani et al.	BMI	Negative (male)
						Repple et al.	BMI / WC	Negative
						Figley et al.	BFP	Negative
						Karlsson et al.	-	N > OV/OB
						Papageorgiou et al.	-	N > OV/OB
						Rahmani et al.	BMI	Positive (male)

S7 Table. Comparison of findings of this work with state of the art that used other statistics. Data is presented in the same manner as Table S4. Abbreviations used: N: normal weight; OV/OB: overweight/obese; BMI: body mass index; AFR: abdominal fat ratio; WC: waist circumference; BFP: body fat percent.
